# Cardiac Computed Tomography in Monitoring Revascularization

**DOI:** 10.3390/jcm12227104

**Published:** 2023-11-15

**Authors:** Elisabetta Tonet, Veronica Amantea, Davide Lapolla, Paolo Assabbi, Alberto Boccadoro, Maria Letizia Berloni, Marco Micillo, Federico Marchini, Serena Chiarello, Alberto Cossu, Gianluca Campo

**Affiliations:** 1 Cardiology Unit, Azienda Ospedaliero Universitaria of Ferrara, 44124 Ferrara, Italy; 2 Radiology Unit, Department of Translational Medicine, University of Ferrara, 44124 Ferrara, Italy

**Keywords:** cardiovascular computed tomography, coronary artery disease, stenting

## Abstract

The use of coronary computed tomography angiography (CCTA) in the setting of stable coronary artery disease is highly recommended for low-risk patients. High-risk patients, such as symptomatic subjects with prior revascularization, are suggested to be investigated with noninvasive functional tests or invasive coronary angiography. CCTA is not considered for these patients because of some well-known CCTA artifacts, such as blooming and motion artifacts. However, new technology has allowed us to obtain images with high spatial resolution, overcoming these well-known limitations of CCTA. Furthermore, the introduction of CT-derived fractional flow reserve and stress CT perfusion has made CCTA a comprehensive examination, including anatomical and functional assessments of coronary plaques. Additionally, CCTA allows for plaque characterization, which has become a cornerstone for the optimization of medical therapy, which is not possible with functional tests. Recent evidence has suggested that CCTA could be used with the aim of monitoring revascularization, both after coronary bypass grafts and percutaneous coronary intervention. With this background information, CCTA can also be considered the exam of choice in subjects with a history of revascularization. The availability of a noninvasive anatomic test for patients with previous coronary revascularization and its possible association with functional assessments in a single exam could play a key role in the follow-up management of these subjects, especially considering the rate of false-positive and negative results of noninvasive functional tests. The present review summarizes the main evidence about CCTA and coronary artery bypass grafts, complex percutaneous coronary intervention, and bioresorbable stent implantation.

## 1. Introduction

It is well-established that cardiac computed tomography angiography (CCTA) has recently played a key role in the area of ischemic heart disease (IHD). For individuals with a low to moderate pre-test risk of coronary artery disease (CAD), the most recent guidelines for chronic coronary syndrome advocate for using CCTA as the primary anatomical test [1]. Regarding acute chest pain, this approach was designed to both confirm and exclude patients in the emergency department when electrocardiograms and laboratory biomarkers did not provide conclusive results [2]. In patients with previous coronary revascularization, both with percutaneous and surgical techniques, the anatomical assessment of coronary arteries has been a prerogative of invasive coronary angiography because of several limitations of CCTA. As a matter of fact, the metallic elements of stented coronary segments lead to a blooming effect, beam-hardening artifacts, and a partial volume effect that makes the assessment of in-stent disease progression challenging. All these factors reduce visualization of the true in-stent lumen, and it has been estimated that 12% of all coronary stents cannot be imaged with adequate diagnostic quality [2]. However, the development of new technology has allowed us to overcome these limitations. The newly established multi-slice CT scan employs multi-row detector array systems permitting a rapid imaging modality allowing views of cardiac structures during one breath hold. Novel CT scanner developments have introduced photon-counting detector technology, which is a sophisticated system improving spatial resolution thanks to a smaller detector scheme when compared with the conventional one [2]. Additionally, the introduction of stress CT perfusion and CT-derived fractional flow reserve (FFR-CT) allows for the functional assessment of coronary stenosis detected with CCTA [3]. The introduction of all this new technology has inspired the use of CCTA for the evaluation of patients already treated with a coronary artery bypass graft (CABG), complex percutaneous coronary intervention (PCI), and a bioresorbable scaffold (BRS) (Graphical abstract). The availability of a noninvasive anatomic test for patients with previous coronary revascularization and its possible association with functional assessment in a single exam could play a key role in the follow-up management of these subjects, especially considering the rate of false-positive and negative results of functional examinations. The present review sought to summarize the current evidence for the use of CCTA in these settings (Table 1).

## 2. New Technologies in CCTA

In recent years, new technological developments have been introduced in the field of CT scanners. Advanced multi-detector CT scanners from newer generations exhibit enhanced spatial and temporal resolution, along with comprehensive heart coverage using wide-detector or dual-source CT. The former offers a coverage area of 16 cm and can capture heart images in a single heartbeat. Dual-source CT can image the heart in about 300 ms. It uses two X-ray tubes and two detectors arranged at 90° angles; this technology allows the reconstruction of images at one quarter of the gantry rotation time, improving temporal resolution. It also shows a good diagnostic image quality in patients with fast heart rates, limiting the use of beta-blockers. Additionally, thinner detectors and faster gantry rotation have also allowed for good image quality in patients with coronary stents and CABG [3].

Photon-counting computed tomography (PCCT) is a recently introduced CT technology. It is based on a new generation of X-ray detectors; they are composed of semiconductor materials that directly convert each X-ray photon into electron-hole pairs. This mechanism enables the counting of photons and their classification into energy levels, avoiding noise at the electronic level. In this way, a substantial spatial resolution improvement can be achieved. Additionally, this technology is related to a decrease in the radiation dose and the amount of contrast media [3,14].

It must be noted that only 49% of significant CCTA stenoses are associated with abnormal invasive fractional flow reserve, such that current recommendations emphasize that patients with a stenosis of 50% or more are recommended to undergo further investigation with a functional test to guide revascularization. The FFR-CT technique has been developed with the aim of obtaining a noninvasive functional assessment of coronary stenosis. It works on the basis that by considering anatomical coronary features and applying computational flow dynamic algorithms, the coronary reply to adenosine administration can be estimated. A patient-specific three-dimensional (3D) anatomic coronary artery model is obtained, and a physiologic model is then derived based on patient-specific inflow and outflow hemodynamic conditions, with the resting myocardial blood flow proportional to the myocardial mass and the mathematical estimation of microvascular resistance. The decreased hyperemic microvascular resistance to adenosine is also predicted, with no need for adenosine administration. In this way, this analysis can forecast the performance of coronary circulation during conditions of maximum hyperemia [3]. FFR-CT does not require additional scan data, and it is associated with fast processing times. The addition of FFR-CT to coronary CTA improves its specificity by evaluating lesion-specific ischemia, enhances its role as a gatekeeper for ICA by decreasing nonobstructive disease at ICA, and offers guidance for revascularization decisions and planning.

Another technique for functional assessment of coronary stenosis is stress CT perfusion. It takes into account that under resting conditions, the coronary circulation maintains a consistent pressure gradient thanks to an autoregulation mechanism; in the presence of a hyperemic stimulus, this autoregulation is disrupted, resulting in reduced myocardial perfusion when coronary stenosis is present. A rest/stress protocol is recommended, using adenosine as a stressor.

Stress CT perfusion images can be obtained using both static and dynamic protocols. The static protocol involves capturing a complete dataset of images throughout the entire cardiac volume during the passage of the contrast medium.

The dynamic protocol, on the other hand, involves capturing multiple datasets that correspond to the contrast kinetics within the cardiac chambers, allowing for the derivation of time-attenuation curves.

This approach enables the assessment of myocardial blood flow in each individual myocardial segment [3].

## 3. Brief Methodological Considerations

A Medline search of full-text articles published in English until October 2023 was performed. Overall, 549 records were identified. The search terms were: ((coronary CTA) OR (cardiac computed tomography) OR (CCTA) OR (CTA) OR (coronary CT)) AND ((left main stent) OR (LM PCI)); ((coronary CTA) OR (cardiac computed tomography) OR (CCTA) OR (CTA) OR (coronary CT)) AND ((bioresorbable scaffold) OR (BRS) OR (bioresorbable stent)); ((coronary CTA) OR (cardiac computed tomography) OR (CCTA) OR (CTA) OR (coronary CT)) AND ((coronary artery bypass graft) OR (CABG)). Only papers published in English-language peer-reviewed journals were selected.

After evaluation of the title and abstract, a total of 32 studies were analyzed as full text.

The quality of selected papers was tested using MINORS criteria. Unblinded reviewers performed the analysis of the full texts for quality assessment. Discrepancies between reviewers have been solved by consensus. The maximum score obtained was 14, and the minimum was 8. We included in the present review only studies obtaining a score of 10. A total of 10 papers were then considered for this overview.

## 4. CCTA after Complex PCI

**Figure 1:**
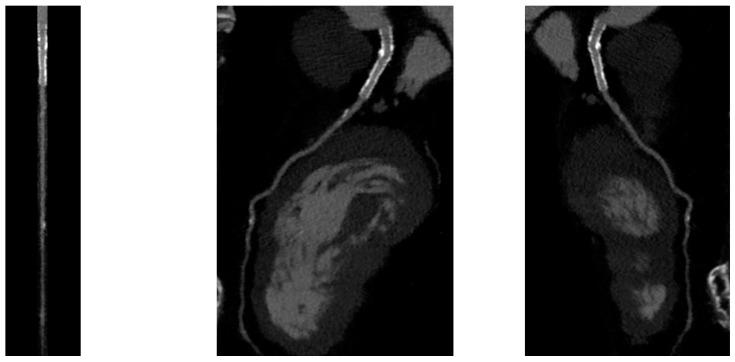
CCTA assessment of left main drug-eluting stent (Lumen image on the **left**, multiplanar reconstruction in the **center** and on the **right**). With a new CT scan, beam-hardening artifacts are reduced, and the stent lumen can be assessed with good performance.

**Figure 2:**
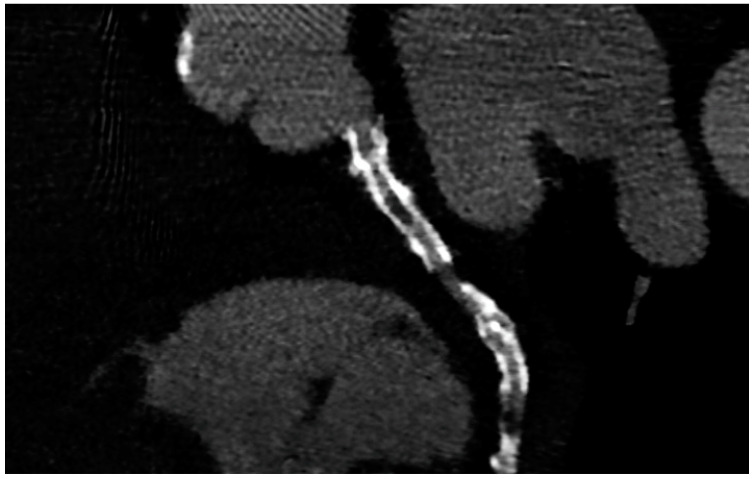
ISR in a stent previously implanted in the proximal segment of the left circumflex artery.

PCI of the left main (LM) improves survival, and in most cases, it is not inferior to surgical revascularization [14]. Intra-stent restenosis (ISR) is a complication of paramount importance, especially in the setting of LM revascularization, because of its relationship to adverse events. With current stents, ISR is due to neo-atherogenesis, which leads to a higher risk of destabilization and stent thrombosis. In the setting of LM, ISR at 15-month follow-up has been revealed to be present in up to 16% of total subjects, requiring invasive revascularization in 7% of cases. Coronary angiography represents the best technique for ISR assessment [14]. Planned Angiography Control (PAC) has been proposed to diagnose and treat ISR promptly, but its benefit remains to be established. An increased rate of percutaneous coronary interventions (PCI) without a reduction in cardiovascular events has been mainly reported. Some technical issues about the use of CCTA in coronary stent imaging have been described, such as the blooming effect, partial volume effect, motion artifacts, and inadequate intravascular contrast enhancement. The blooming effect is the most important issue, corresponding to a phenomenon in which stent struts appear thicker, causing an underestimation of stent lumen. However, with new technology development, CCTA provides a precise, noninvasive reconstruction of the coronary tree and may offer an alternative to invasive coronary angiography [15]. Figure 1 shows three multiplanar reconstructions of LM and left anterior descending with a drug-eluting stent previously implanted: struts of the stent appear clearly detectable, the stent seems to be well-positioned, and regarding stent lumen, it can be noted that there is good opacification and no evidence of ISR. Medium and distal tracts of the left anterior descending seem to be free from plaque proliferation and/or stenosis. Figure 2, instead, shows an ISR of a stent implanted in the proximal segment of the left circumflex coronary artery. Therefore, Figure 1 and Figure 2 demonstrate the feasibility of LM and proximal segment stent assessment by CCTA. Its use in the PAC setting has been investigated and may provide relevant advantages as it is a noninvasive examination. There are three different methods to determine the degree of ISR with CCTA: qualitative, semi-quantitative, and quantitative. The first technique provides that significant ISR (reduction of luminal diameter >50%) is visually detected: ISR is identified as a hypodense layer between the struts and the lumen. The second method is characterized by a four-point scale where 1 corresponds to the patency of the stent and 4 results in stent occlusion. Finally, the third technique provides that the percentage of stenosis is calculated as the ratio between diameters in the short axis of the narrowest stent lumen and of the proximal and distal reference segments [15]. Roura G et al. evaluated the agreement between CCTA and intravascular ultrasound (IVUS) to assess in-stent lumen diameters and lumen area of LM stents: the study highlighted a good agreement between the two techniques so that CCTA can be considered to analyze LM ISR [4]. A study by Van Mieghem CAG et al. assessed the performance of CCTA in the analysis of LM stenting: they enrolled 74 patients scheduled for follow-up coronary angiography after LM stenting, and they performed CCTA before coronary angiography. The study demonstrated that the accuracy of CCTA for detecting LM ISR was 98%; in particular, diagnostic accuracy was 98% both for patients with stented LM and with distal LM bifurcation lesions and only one side branch treated [5]. In subjects with complex bifurcation stenting (i.e., LM and both major side branches), the reliability of CCTA was 83%. The low number of false-positive scans leading to unnecessary diagnostic coronary angiograms should be acceptable, taking into account the potentially serious consequences of LM ISR [5]. Furthermore, the study underlined that the evaluation of stent diameter and area by CCTA had a good correlation with IVUS assessment. One of the most important issues arising from this study was the high radiation dose required for the analysis of stents by CCTA. However, as previously reported, new scanner developments (i.e., dual-source CT scanners) reduced patient dose.

Although LM PCI is considered a complex procedure, the assessment of LM stenting by CCTA represents the “best-case scenario” for imagers. Stents implanted in the LM are typically large, and LM runs in an axial plane that corresponds to the scan direction, making it relatively free from motion artifacts. Additionally, it is essential to note that new technologies, such as Photon-Counting CCTA, are currently available in selected centers. These technologies allow for a more detailed assessment of stented segments, providing a better estimation of ISR.

Despite these advancements, there is currently a lack of data regarding the use of CCTA in the follow-up of LM stenting from randomized trials. The “Angiographic control vs. ischemia-driven management of Patients undergoing percutaneous revascularization of the Unprotected Left main coronary artery with Second-generation drug-Eluting stents (PULSE) trial” (NCT04144881) is an ongoing prospective, multicenter, randomized trial aiming to determine whether a PAC strategy based on CCTA is superior to ischemia and symptom-driven conservative management approach in reducing major adverse cardiac events (MACE) and target lesion revascularization at 18 months. The results of the PULSE trial could define the role of CCTA in this particular clinical setting.

## 5. CCTA after Scaffold Implantation

**Figure 3:**
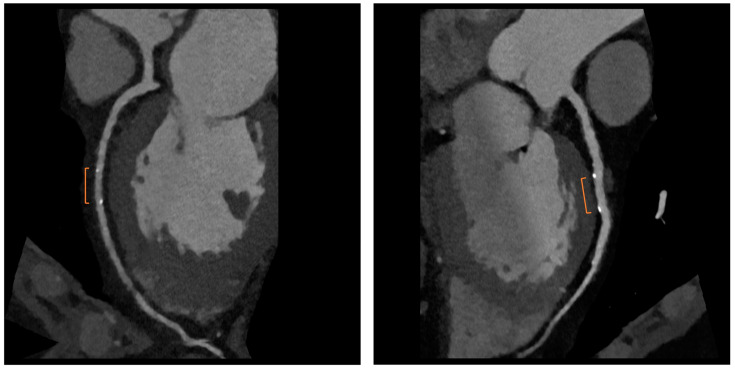
CCTA assessment of BRS. Orange brackets highlight the two markers of BRSs whose struts result in complete reabsorption. The vessel is also well analyzed in the scaffolded segment.

**Figure 4:**
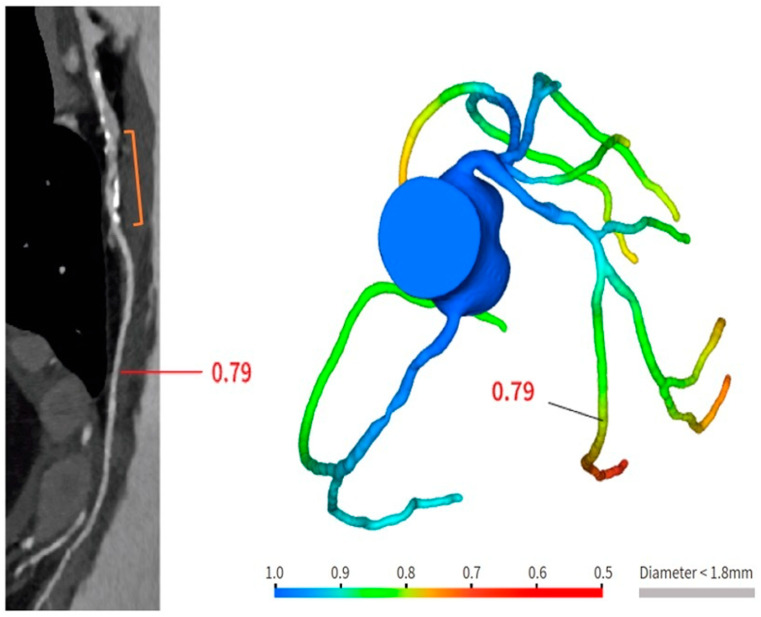
Anatomical and functional assessment of ISR in a scaffold (orange bracket). ISR appears to be characterized by a major calcific part, and the stenosis results were significant under the FFR-CT assessment. FFR-CT was performed with DeepVessel FFR (DVFFR) software (Keya Medical, Seattle, WA, USA).

Bioresorbable scaffolds (BRS) were designed to combine the short-term advantages of permanent stents with the long-term benefit of complete reabsorption, facilitating the restoration of vasomotor and endothelial function. This technology helps prevent prolonged inflammation, maintains the integrity of distal bypass grafting sites, and allows unimpeded future vessel imaging. Despite the promising theoretical benefits of BRS, the initial generation of BRS devices exhibited higher rates of stent thrombosis in comparison to other stents [16]. Newer generation devices appear to present a viable alternative to drug-eluting stents in the management of acute coronary syndromes (ACS) for several reasons: their different composition when compared to first-generation BRS, the optimized deployment technique, and the lesion selection. Notably, ACS lesions show specific characteristics based on the pathophysiology of the disease [16]. Thus, various factors create favorable conditions for BRS implantation in ACS patients, including the vulnerable nature of the plaque, minimal calcification, the presence of a thrombus, and the relative youth of patients. BRS is radiolucent except for two metallic radio-opaque markers located at both extremities. This design feature aids in visualization during imaging procedures, ensuring accurate placement and monitoring of the scaffold. Thus, CCTA can delineate the contours of the scaffolded segment: markers easily enable the location of where BRS was implanted, and they can be distinguished from calcification because of the difference in attenuation [17]. Figure 3 shows a CCTA analysis of scaffolded coronary segments: as highlighted by orange brackets, there is evidence of two little markers of the scaffold that appear completely reabsorbed: indeed, no struts are detectable, and the vessel lumen can be analyzed in depth also in the scaffolded part with no evidence of plaque proliferation. The diagnostic accuracy of coronary CT angiography in poli-LLA (poly-L-lactide) Everolimus scaffold was studied in the ABSORB II study (A Bioresorbable Everolimus-Eluting Scaffold Versus a Metallic Everolimus-Eluting Stent II) [6]. The study provided the randomization of enrolled patients to receive treatment with BRS or drug-eluting stent. At the 3-year follow-up, patients treated with BRS underwent coronary angiography with intravascular ultrasound (IVUS) evaluation and CCTA. The study demonstrated that the CCTA diagnostic accuracy for detecting in-scaffold obstruction and luminal dimensions was similar to invasive coronary angiography (ICA) and IVUS. Analyzing scaffold segments, the sensitivity, specificity, and negative predictive values were 71%, 82%, and 97%, respectively, using IVUS as a reference. One limitation of this study was its use of a 3-year follow-up period, which did not address the crucial question of assessing the occurrence of restenosis within the initial 12 months. It is during this period that most restenosis events occur, coinciding with the presence of BRS with thicker struts in place [18]. Salinas P et al. performed the first case series of Magnesium bioresorbable scaffold investigated with CCTA at 1 year of follow-up [7]. The CCTA in-scaffold percentage diameter stenosis and area stenosis were 22% and 39%, respectively, underlying plaque growth. Additionally, performing plaque characterization, the segments treated with RMS showed that the most common component of the plaque was the fibrous one (69% of the cases), suggesting that RMS allows for the stabilization of culprit lesions [7]. Furthermore, anatomical findings can be combined with noninvasive fractional flow reserve derived from CCTA (FFR-CT) to distinguish the presence or absence of flow-limiting disease [19]. A study by Tonet E et al. investigated the performance of CCTA and FFR-CT in 26 patients treated with Magnesium bioresorbable scaffold: all patients underwent CCTA 18 months after BRS implantation. The left anterior descending artery was the most commonly affected vessel. CCTA revealed patent scaffolded segments, with complete strut reabsorption observed in 93% of cases. FFR-CT demonstrated to be feasible in scaffolded segments with a median value of 0.88 [0.81–0.91]. Figure 4 shows a case from the above-reported study: BRS (orange bracket) appears to be characterized by plaque proliferation with a prevalent calcific component. FFR-CT analysis highlighted a significant stenosis related to the plaque. In conclusion, these results suggest that CCTA plus FFR-CT is a valuable noninvasive tool for the assessment of coronary arteries in subjects treated with BRS. Scaffolded segments can be easily distinguished, allowing for quantitative measurements and the calculation of noninvasive FFR. The analysis also indicates a tendency to observe plaque stabilization in the scaffolded segments with fibrosis and calcium [8]. However, further evidence is needed in this setting of BRS patients.

## 6. CCTA Post-CABG

**Figure 5:**
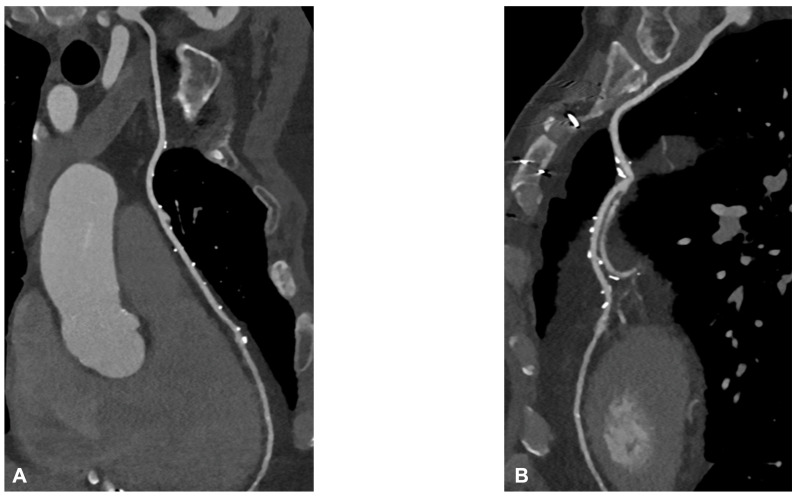
In (**A**,**B**), two multiplanar reconstructed images of the left internal mammary artery CABG on the left anterior descending coronary artery. There are no signs of degenerative disease with the patency of grafts.

**Figure 6:**
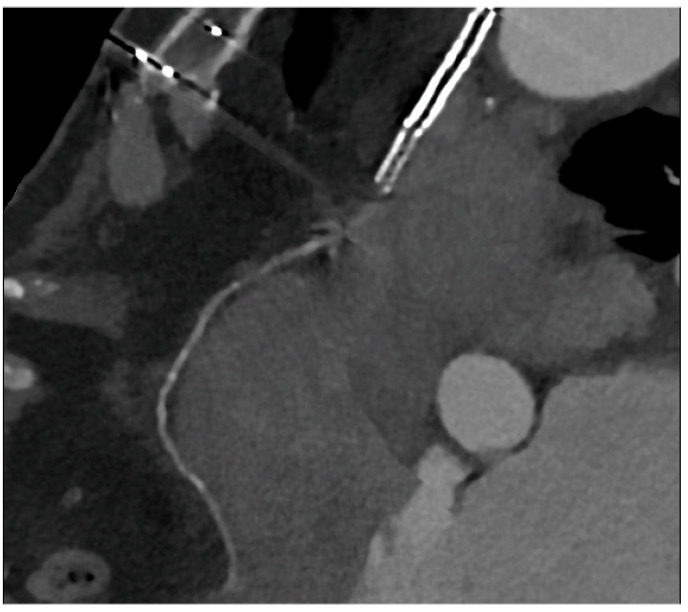
An occluded saphenous vein graft to the right coronary artery. The graft was previously treated with a stent, which appears occluded.

Graft failure remains a significant concern, with reported occurrence rates ranging from 3% to 10%. Data from the literature underlined that the occlusion of the internal mammary artery within the initial year following surgery has a prevalence of 5.7% in men and 3.4% in women [20]. The saphenous vein graft showed a lower patency rate compared with the internal mammary artery. Typically, saphenous grafts exhibit occlusion rates of 12% within the first six months after surgery, which increases to 25% after five years. Subsequently, the patency rate declines to 50% at 15 years or more post-surgery [21]. Angina may be associated with graft failure and occlusion, but within the first five years following surgery, in 50% of cases, angina is linked to the progression of native coronary artery obstruction [22]. Graft failure can lead to several issues, such as refractory angina, myocardial ischemia, arrhythmias, low cardiac output, and fatal cardiac failure, emphasizing the importance of ensuring graft patency during and after surgery to prevent complications. To address this issue, several techniques have been provided to evaluate graft patency after coronary artery bypass graft (CABG) surgery [23]. Coronary angiography is currently the gold standard for assessing the status of CABG; however, this is an invasive method, and it has a certain number of complications. Engagement and visualization of venous and arterial bypass grafts frequently prolong procedure time and are associated with larger contrast use, increased radiation exposure, and higher risk of embolization and dissection during catheter manipulation [11]. Therefore, there is a need for a noninvasive method with good diagnostic accuracy for the follow-up of CABG patients. The 2010 multi-societal Appropriate Use Criteria (AUC) defined coronary Computed Tomography Angiography (CTA) as “Appropriate” for the evaluation of CABG patency in patients with ischemic symptoms [24]. Surgical grafts are ideal vessels to be assessed by CCTA because of their large diameter, low incidence of severe calcifications, and less influence by heart movements when compared to native coronary arteries. Figure 5A,B and Figure 6 show some examples of the CABG assessment by CCTA. In Figure 5, the left internal mammary artery was used, and an anastomosis with the native left coronary artery was created. Figure 5 highlights the feasibility of CCTA in CABG patients, with good diagnostic accuracy of grafts that appear free from plaque proliferation. The distal part of native coronary arteries appears patent with a good opacification by contrast medium. Figure 6 shows an occluded saphenous vein graft previously treated with stenting. In a study that included symptomatic patients who had undergone CABG surgery approximately 10 ± 5 years prior and underwent CCTA, Malagutti et al. demonstrated that CCTA exhibited a sensitivity of 100% and a specificity of 98.3% in detecting graft patency when compared to ICA. Additionally, their findings indicated that overestimation of obstruction was more likely in native coronary arteries, particularly in the presence of calcification [9]. In a meta-analysis by Barbero et al., which focused on patients experiencing angina or suspected symptoms of myocardial infarction following CABG, the study examined the sensitivity and specificity of 64-slice CT in detecting graft occlusion and stenosis exceeding 50%. The sensitivity and specificity for identifying any coronary artery bypass graft with stenosis greater than 50% were found to be 0.98 (95% CI: 0.97–0.99) and 0.98 (95% CI: 0.96–0.98), respectively. These observations remained consistent regardless of age and were applicable to both arterial and venous conduits, resulting in an area under the curve of 0.99 were independent of age and consistent in both arterial and venous conduits resulting in an area under the curve of 0.99 [10]. A comparative study between CCTA and ICA by Weustink et al. involving symptomatic post-CABG patients concluded that CCTA demonstrated a diagnostic accuracy of 100% in identifying or ruling out significant stenosis in grafts. The specificity, sensitivity, PPV, and NPV all yielded 100% accuracy in the detection of significant stenosis. As a result, they concluded that CCTA was valuable and exhibited high diagnostic accuracy for identifying post-CABG significant stenosis or graft occlusion [11]. Although CCTA is highly accurate for bypass grafts, the evaluation of native coronary arteries in patients with prior CABG can be challenging due to the diffuse and severe nature of underlying coronary artery disease. For example, the sensitivity for detection of stenosis ≥ 50% in recipient and nongrafted vessels is typically lower (83–90%) in patients with CABG than in patients without prior CABG. However, there are limitations associated with CCTA, which encompass its incapability to identify stenosis or blockages in heavily calcified coronary arteries, the tendency to overestimate stenosis due to calcification, challenges in visualizing distal anastomosis, image quality issues stemming from artifacts caused by hemostatic metal clips, difficulties when patients cannot hold their breath, rapid heart rate, atrial fibrillation, and residual coronary motion [25]. However, the novel whole-heart coverage CT scanners allow the avoidance of some of the above-mentioned limitations: a study by Mushtaq S et al. showed that CCTA successfully interpreted 100% of the bypass grafts. When compared to ICA, CCTA exhibited the ability to identify occlusion and significant stenosis in all CABG segments, with sensitivity, specificity, PPV, and NPV of 100% each for the grafts. With these results, the study concluded that last-generation whole-heart coverage CT scanners can assess bypass grafts with very good interpretability and a lower level of radiation [12].

Presently, the randomized trial by Jones DA et al. investigated the advantages of performing CCTA before ICA in patients with CABG: the study demonstrated that the cohort undergoing CCTA showed reduced procedure time and contrast-induced nephropathy [13]. Further data about this topic will be provided by the GREECE (Computed Tomography Guided Invasive Coronary Angiography in Patients with a Previous Coronary Artery Bypass Graft Surgery) randomized trial: it is recruiting patients to compare radiation and contrast dosage between individuals with and without prior CCTA when undergoing diagnostic ICA [26].

Regarding risk assessment, numerous studies have highlighted the value of CCTA for long-term risk stratification through the classification of protected and unprotected coronary territories based on graft patency and obstructive native vessel disease [27]. Lastly, findings from the CT-RECTOR (Computed Tomography Registry of Chronic Total Occlusion Revascularization) registry have suggested that performing CCTA before revascularization of a chronic total coronary occlusion (CTO) can predict effective guidewire crossing and optimize procedural times [28,29]. This is particularly significant given that CTOs are observed in over 50% of post-CABG patients referred to the catheterization laboratory.

Concerning functional CT modalities such as CT perfusion and CT-based fractional flow reserve (CT-FFR), there is limited data available for CABG patients in published studies, and thus, their additional value remains to be established [30]. In light of this, the current AHA/ACC/ASE/CHEST/SAEM/SCCT/SCMR Guideline for the Evaluation and Diagnosis of Chest Pain recommends CCTA for post-CABG patients with a level of evidence of 2A [31,32]. In the end, the decision to perform CCTA may depend on the clinical question. On the one hand, when aiming for the assessment of graft patency, CCTA is an appropriate and well-validated examination. On the other hand, when evaluating native coronary arteries, attention to image acquisition to ensure high image quality using CCTA is crucial, and functional testing should be considered. Furthermore, performing CCTA to visualize other structures before re-doing cardiac surgery is advisable.

## 7. Conclusions

The contentious debate regarding whether anatomical or functional assessment is superior for patients with coronary artery disease has been less frequently explored in individuals with a history of coronary revascularization. The high-risk profile of this type of patient has led to a preference for a functional test in the case of recurrent symptoms. However, in recent years, CCTA has shown advancements in technology that have delivered substantial benefits through enhancements in image quality and reduction of overall radiation exposure so that CCTA represents a promising technique in the assessment of patients previously treated with CABG, complex PCI such as LM PCI, and BRS. The advantages of using CCTA in these settings are several: (a) it is a noninvasive assessment, so it is free from percutaneous angiogram risks; (b) the acquisition is faster than other noninvasive imaging modalities such as stress cardiac magnetic resonance or stress echocardiography; (c) it allows plaque characterization in order to optimize medical therapy; (d) it could be related to a lower amount of unnecessary coronary angiography when compared to symptom-driven strategy; (e) functional assessment can be added to the anatomical one; (f) CCTA can predict PCI results using virtual PCI software, which allows the choice of the best stenting diameter and length to obtain the best final result; (g) stress CTP is able to evaluate myocardial perfusion and to estimate myocardial blood flow. With this background, CCTA is expected to become the exam of choice in the whole spectrum of stable coronary artery disease.

## Figures and Tables

**Table 1:** Summary of main evidence available till now about CCTA in patients with previous revascularization. For abbreviations, see main text.

**CCTA after Complex PCI**	
**Roura G et al., 2013** [4]	Good agreement between CCTA and IVUS in the assessment of in-stent lumen diameters and lumen area of LM stents.
**Van Mieghem CAG et al., 2006** [5]	CCTA showed a good performance in pointing out LM ISR with an accuracy of 98% (ICA as reference). Good correlation with IVUS in the evaluation of stent diameter and area.
**CCTA after scaffold implantation**	
**Collet C et al., 2018** [6]	At 3-year follow-up after BRS implantation, CCTA diagnostic accuracy for detecting in-scaffold obstruction and luminal dimensions was similar compared with invasive coronary angiography (ICA) and IVUS. Analyzing scaffold segments, the sensitivity, specificity, and negative predictive values were 71%, 82%, and 97%, respectively, using IVUS as a reference.
**Salinas P et al., 2020** [7]	CCTA is feasible in scaffolded coronary segments.
**Tonet E et al., 2022** [8]	CCTA and FFR-CT in 26 patients treated with Magnesium bioresorbable scaffold. FFR-CT demonstrated to be feasible in scaffolded segments.
**CCTA post-CABG**	
**Malagutti P et al., 2007** [9]	CCTA exhibited a sensitivity of 100% and a specificity of 98.3% in detecting graft patency when compared to ICA. Additionally, their findings indicated that overestimation of obstruction was more likely in native coronary arteries, particularly in the presence of calcification.
**Barbero U et al., 2016** [10]	The sensitivity and specificity of CCTA for identifying any coronary artery bypass graft with stenosis greater than 50% were found to be 0.98 (95% CI: 0.97–0.99) and 0.98 (95% CI: 0.96–0.98), respectively.
**Weustink AC et al., 2010** [11]	CCTA showed a diagnostic accuracy of 100% in identifying or ruling out significant stenosis in grafts. The specificity, sensitivity, PPV, and NPV all yielded 100% accuracy in the detection of significant stenosis.
**Mushtaq S et al., 2020** [12]	CCTA successfully interpreted 100% of the bypass grafts. When compared to ICA, CCTA exhibited the ability to identify occlusion and significant stenosis in all CABG segments, with a sensitivity, specificity, PPV, and NPV of 100% each for the grafts.
**Jones DA et al., 2023** [13]	In a randomized cohort, CCTA before ICA leads to reductions in procedure time and contrast-induced nephropathy.

## Data Availability

Not applicable.
